# Comparison of Two Hybrid Models for Forecasting the Incidence of Hemorrhagic Fever with Renal Syndrome in Jiangsu Province, China

**DOI:** 10.1371/journal.pone.0135492

**Published:** 2015-08-13

**Authors:** Wei Wu, Junqiao Guo, Shuyi An, Peng Guan, Yangwu Ren, Linzi Xia, Baosen Zhou

**Affiliations:** 1 Department of Epidemiology, School of Public Health, China Medical University, Shenyang, PR China; 2 Liaoning Provincial Center for Disease Control and Prevention, Shenyang, PR China; The University of Tokyo, JAPAN

## Abstract

**Background:**

Cases of hemorrhagic fever with renal syndrome (HFRS) are widely distributed in eastern Asia, especially in China, Russia, and Korea. It is proved to be a difficult task to eliminate HFRS completely because of the diverse animal reservoirs and effects of global warming. Reliable forecasting is useful for the prevention and control of HFRS.

**Methods:**

Two hybrid models, one composed of nonlinear autoregressive neural network (NARNN) and autoregressive integrated moving average (ARIMA) the other composed of generalized regression neural network (GRNN) and ARIMA were constructed to predict the incidence of HFRS in the future one year. Performances of the two hybrid models were compared with ARIMA model.

**Results:**

The ARIMA, ARIMA-NARNN ARIMA-GRNN model fitted and predicted the seasonal fluctuation well. Among the three models, the mean square error (MSE), mean absolute error (MAE) and mean absolute percentage error (MAPE) of ARIMA-NARNN hybrid model was the lowest both in modeling stage and forecasting stage. As for the ARIMA-GRNN hybrid model, the MSE, MAE and MAPE of modeling performance and the MSE and MAE of forecasting performance were less than the ARIMA model, but the MAPE of forecasting performance did not improve.

**Conclusion:**

Developing and applying the ARIMA-NARNN hybrid model is an effective method to make us better understand the epidemic characteristics of HFRS and could be helpful to the prevention and control of HFRS.

## Introduction

Hemorrhagic fever with renal syndrome (HFRS) is a rodent-borne disease caused by Hantaviruses from the family Bunyaviridae. Cases of HFRS are widely distributed in eastern Asia, particularly in China, Russia, and Korea. China accounts for about 90% of total reported cases worldwide [1]. Nowadays, HFRS is endemic in 28 of 31 provinces in mainland China [2]. Jiangsu province, a highly developed coastal province, is one of the most severely affected provinces in China [3]. Although the government has adopted effective and comprehensive ways to control the transmission of HFRS [4], there are still some factors such as diverse animal reservoirs and effects of global warming will influence control effects. Therefore, it is proved to be a difficult task to eliminate HFRS completely.

Statistical models have been widely used in infectious disease forecasting. Reliable forecasting can make people better understand the epidemic characteristics of infectious disease and prepare for intervention measures in advance. Nowadays, statistical models including autoregressive integrated moving average (ARIMA) model [5–7] and linear regression model [8, 9] have been used to predict the incidence of HFRS. However, these linear assumption models cannot always fit complex real-world problems well which generally exhibit some nonlinear characteristics. Models based on artificial neural networks (ANNs) can effectively extract nonlinear relationships in the data [10]. However, ANNs cannot handle both linear and nonlinear patterns equally well [11]. Therefore, many researchers have developed some hybrid models combining ARIMA model with ANNs to forecast the incidence or prevalence of infectious disease and achieved good effects. Hybrid model combining ARIMA model and nonlinear autoregressive neural network (NARNN) model has been used to forecast the prevalence of schistosomiasis [12] and the incidence cases of hand-foot-mouth disease [13]; Hybrid model combining ARIMA model and generalized regression neural network (GRNN) has been used to predict incidence of bacillary dysentery [14] and tuberculosis [15, 16]. All of these hybrid models have higher quality prediction accuracy than ARIMA model alone. There is nearly no article devoted to a study of prediction of HFRS based on these hybrid models. Therefore, we intended to create two hybrid models, one composed of NARNN and ARIAM the other composed of GRNN and ARIMA to predict HFRS incidence in Jiangsu province, China. Performances of the two hybrid models were compared with ARIMA model. The aim of this study is to explore the optimal model and to describe the future trend of HFRS more accurate. This will be useful for the prevention and control of HFRS.

## Materials and Methods

### Study Area and Data Collection

Jiangsu is located at 116.60°~121.67° east longitude and 31.01°~34.89° north latitude on the central coast of China and has an area of 102.6 thousand square kilometers [17]. Jiangsu borders Shandong in the north, Anhui to the west, and Zhejiang and Shanghai to the south. Jiangsu has a coastline of over 1,000 kilometers along the Yellow Sea, and the Yangtze River passes through the southern part of the province. Most of Jiangsu has a humid subtropical climate, beginning to transition into a humid continental climate in the far north. Rain falls frequently between spring and summer, typhoons with rainstorms occur in late summer and early autumn.

**Fig 1:**
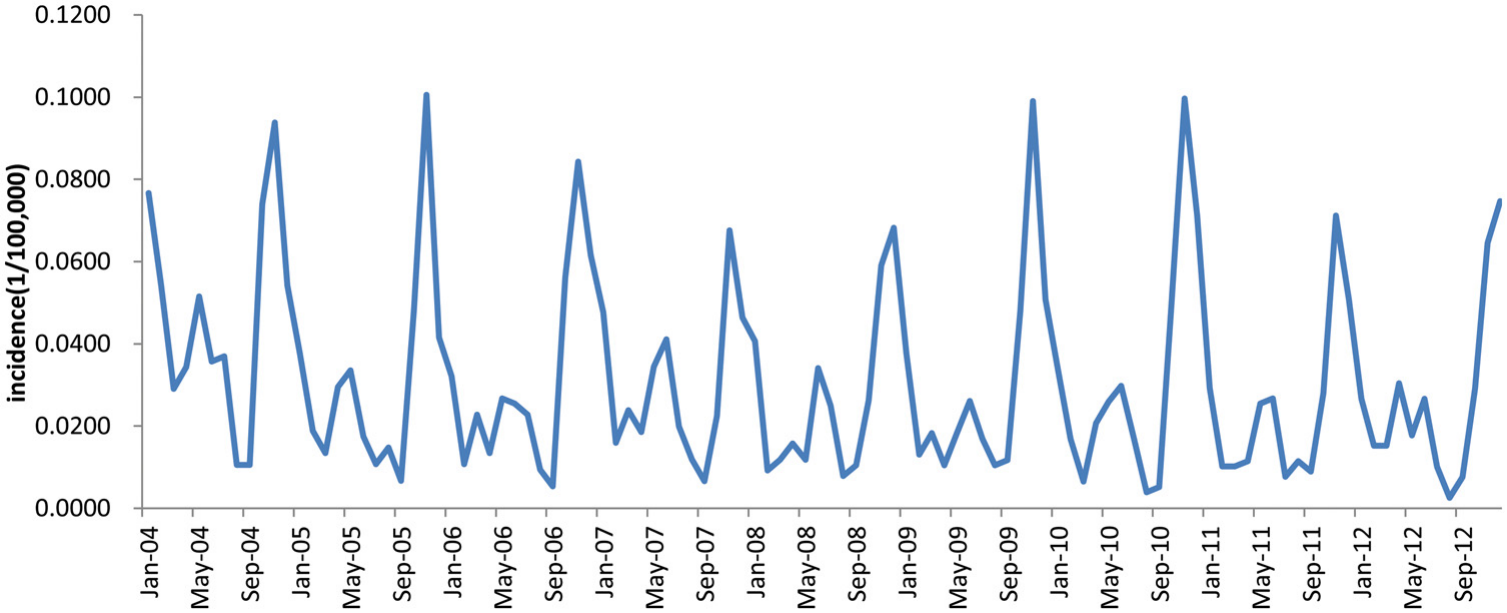
Monthly HFRS incidence series of Jiangsu province in China from January 2004 to December 2012.

HFRS is one of Nationally Notifiable Infectious Diseases in China. The monthly data of reported HFRS incidence in Jiangsu Province from January 2004 to December 2012 was obtained from the Chinese National Surveillance System (CNSS) established in 2004. The original time series data is presented in S1 File. The time series data of HFRS incidence in Jiangsu Province showed an obvious seasonality trend, with higher incidence rates in spring and autumn-winter seasons (Fig 1). In order to compare the modeling and forecasting performances of two hybrid models and ARIMA model, we divided the data set into two parts. The data set between January 2004 and December 2011 was used as the modeling data set, while the data set between January 2012 and December 2012 was used as the forecasting data set. Ethical statement was not necessary because the data are for public access secondary data.

### ARIMA Model Construction

ARIMA model is a traditional method to study the time series data. Because there was a strong seasonality trend in this study, therefore we constructed a seasonal ARIMA (p,d,q)×(P,D,Q)_s_ model. The model was defined with the number of auto-regressive lags p, moving-average lags q, seasonal auto-regressive lags P, seasonal moving-average lags Q, differences and seasonal differences d and D, and the length of the seasonal period s. Augmented Dickey-fuller Unit Root (ADF) test was applied to estimate the stationary of the time series with Eviews 8.0 (http://www.eviews.com). If the series is not stationary, differencing can be used to transform it into a stationary series. The Box and Jenkins strategy was used construct the seasonal ARIMA model. The conditional Least Squares method was applied to estimate the model parameters. R square, Stationary R square, root mean square error (RMSE), normalized Bayesian Information Criterion (BIC) and Ljung-Box Q test was used to compare the goodness-of-fit among ARIMA models.

### ARIMA-GRNN Model Construction

ANNs are one of the artificial intelligence techniques and have been used widely in fitting nonlinear models. ANNs can gain the regularity by training the known data and then predict the unknown data. Among various artificial neural network models, GRNN model is a universal approximator for smooth functions based on nonlinear regression theory [15]. GRNN model was devised by Speckt, has four layers: the input layer, pattern layer, summation layer, and output layer. The network architectures of GRNN have been previously mentioned [16, 18]. After ARIMA model established, we could obtain the estimated monthly incidence of HFRS. Since the ARIMA model had been used to analyze the linear part of the actual data, the residuals should contain nonlinear relationships [15]. In order to include more effective and useful information in GRNN model, it is necessary to use the time values as one input of GRNN especially when there was a strong seasonality trend. In this study, the estimated monthly incidence values of ARIMA and corresponding time values were used as two inputs of GRNN model, while the actual monthly incidence values were used as the output of GRNN model. Then we can capture the nonlinear component through this hybrid model. The performance of the GRNN mainly depends on the spread factor [19]. The selection of optimal spread factor needs a certain amount of trial. The spread was chosen with the method proposed by Specht [20]. We selected two samples randomly in the modeling data set as testing samples; the other samples were used to fit the GRNN model.

### ARIMA-NARNN Model Construction

In this section, we first generated the residual series with formula: $e_{t}=y_{t}-{\hat{L}}_{t}$. Where *e*_*t*_ is the residual, *y*_*t*_ is the actual monthly incidence and ${\hat{L}}_{t}$ is the predicted monthly incidence by the ARIMA model at time t. According to the principle of NARNN model, there is only one series involved and the series will be dealt as time series automatically. The future values of a time series are predicted only from past values of that series. Then the residual series was used to construct the NARNN model in this study. NARNN is a dynamic neural network based on the back-propagation neural network with the feedback layers to approximate the non-linear function [21]. There is a default tan-sigmoid transfer function in the hidden layer and a linear transfer function in the output layer. A feedback connection from the network output is used as one input of this network. After the network has been trained, this feedback connection can be closed. For each of these inputs, there is a tapped delay line to store previous values. In the course of assigning the network architecture for a NARNN, we should select the delays associated with each tapped delay line, and also the number of hidden layer neurons by trial and error. The modeling data set was randomly divided into three parts, the training subset used to train the network, the validation subset used to stop training before over fitting, the testing subset used as a completely independent test of network generalization the study [12]. In this study, the modeling data set was randomly divided, with 90% used for training, 5% for validation and 5% for testing. The network used the default Levenberg-Marquardt algorithm for training. The error autocorrelation plot, the time series response plot and the correlation coefficient (R) were analyzed to choose the optimal model [12]. All of the training was done in open loop, including the validation and testing steps. After the network was trained, it was transformed to closed loop for multistep-ahead prediction. Based on the adjusted residuals, we could obtain the expected monthly incidence of HFRS with equation: ${\hat{y}}_{t}={\hat{L}}_{t}+{\hat{N}}_{t}$. Where ${\hat{y}}_{t}$ is the expected monthly incidence, ${\hat{L}}_{t}$ is the predicted monthly incidence by the ARIMA model and ${\hat{N}}_{t}$ is the residual adjusted by NARNN model at time t.

### Model Evaluation Index

The mean square error (MSE), mean absolute error (MAE) and mean absolute percentage error (MAPE) were selected as the measures of evaluation of ARIMA, ARIMA-GRNN and ARIMA-NARNN models.

$$\text{MSE}=\frac{1}{n}\overset{n}{\underset{t=1}{\sum }}{\left(y_{t}-{\hat{y}}_{t}\right)}^{2}$$

$$\text{MAE}=\frac{1}{n}\overset{n}{\underset{t=1}{\sum }}\left|y_{t}-{\hat{y}}_{t}\right|$$

$$\text{MAPE}=\frac{1}{n}\overset{n}{\underset{t=1}{\sum }}\frac{\left|y_{t}-{\hat{y}}_{t}\right|}{y_{t}}$$

### Data Analyses Using Computer Software

The ARIMA model was performed with SPSS 22.0. Neural Network Toolbox of MATLAB provides functions and apps for modeling complex nonlinear systems that are not easily modeled with a closed-form equation. The ARIMA-GRNN model and ARIMA-NARNN model were constructed with the Neural Network Toolbox in MATLAB R2014b. The MSE, MAE and MAPE were computed with Microsoft Excel spreadsheets.

## Results

### The Best-fitting ARIMA Model

**Fig 2:**
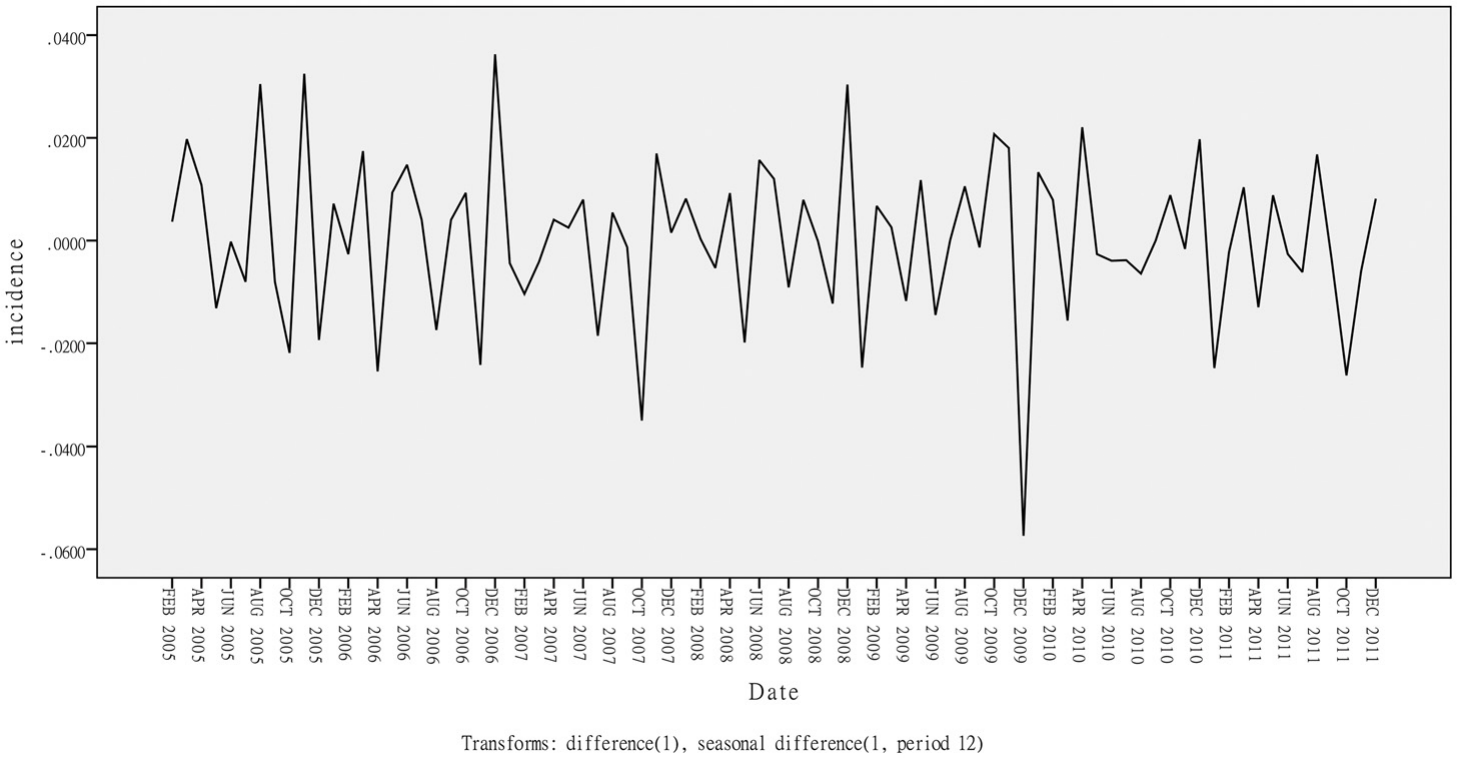
Transformed time series of monthly HFRS incidence. A first-order regular difference and a first seasonal difference were used to transform the raw series into a stationary one.

In the ARIMA analysis, the data set between January 2004 and December 2011 was used to construct model. After the first-order regular difference and the first seasonal difference (Fig 2) the time series was stationary (ADF test: t = -3.0656, P = 0.0026). A series of candidate models were tested. The models were eliminated if they could not pass the parameter test or residual test. At last, three models remained: ARIMA (0,1,1)×(0,1,1)_12_, ARIMA (0,1,1)×(1,1,0)_12_ and ARIMA (1,1,0)×(1,1,0)_12_. Comparison of three candidate models are displayed in Table 1. The best model generated from the modeling data set was ARIMA (0,1,1)×(0,1,1)_12_ according to the goodness-of-fit (R^2^ = 0.7327, stationary R^2^ = 0.4392, RMSE = 0.0119, normalized BIC = -8.7533, Ljung-Box Q statistics = 15.8386, *P* = 0.4643). The parameter estimates of ARIMA (0,1,1)×(0,1,1)_12_ are shown in Table 2. The incidence values from January 2012 to December 2012 were forecasted with the constructed ARIMA (0,1,1)×(0,1,1)_12_ model.

**Table 1:** Comparison of three candidate ARIMA models.

Model	R^2^	Stationary R^2^	RMSE	BIC	Ljung-Box test	
					Q statistics	P value
ARIMA (0,1,1)×(0,1,1)_12_	0.7327	0.4392	0.0119	-8.7533	15.8386	0.4643
ARIMA (0,1,1)×(1,1,0)_12_	0.7139	0.3998	0.0123	-8.6853	15.7424	0.4710
ARIMA (1,1,0)×(1,1,0)_12_	0.6614	0.2896	0.0134	-8.5168	23.3827	0.1039

**Table 2:** Parameter estimates and their testing resulting of the final seasonal ARIMA (0,1,1)×(0,1,1)_12_ model.

Model parameter	Estimate	Standard error	t statistic	P value
MA1	0.7462	0.0827	9.0182	0.0000
Seasonal MA1	0.7111	0.1336	5.3209	0.0000

### ARIMA-GRNN Hybrid Model

**Fig 3:**
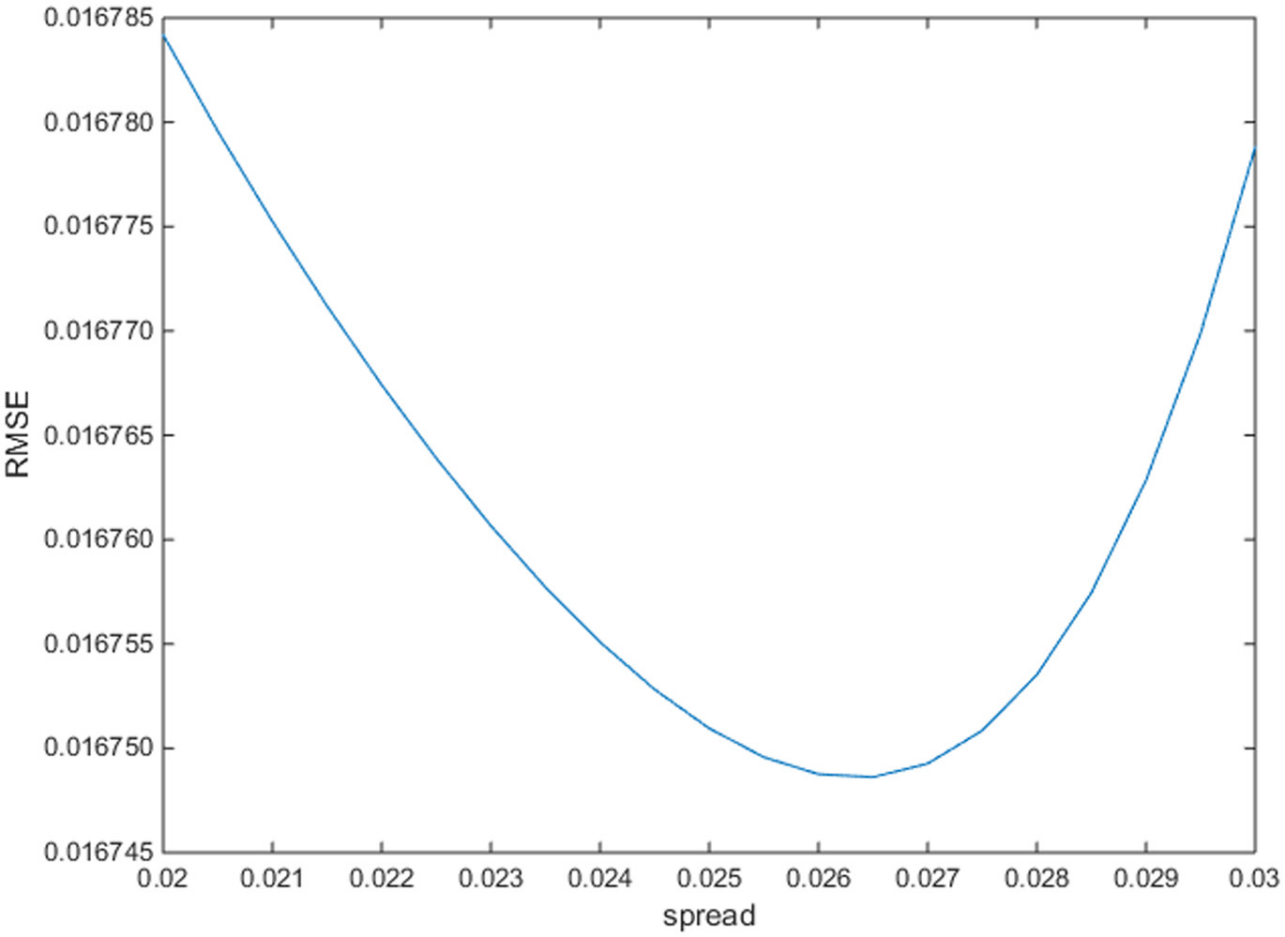
The selection of the optimal spread of the ARIMA-GRNN hybrid model. When the spread was 0.006, the RMSE for the testing samples is the least.

In order to construct the ARIMA-GRNN hybrid model, the predicted monthly incidence values of HFRS from ARIMA and corresponding time values were used as the inputs of GRNN model, while the actual monthly incidence values were used as the target data. There were thirteen samples lost because of the first-order regular difference and the first seasonal difference. Therefore, the data between February 2005 and December 2011 was used to construct GRNN model. The data of June 2007 and November 2009 were randomly chosen as the testing samples to determine the optimal spread. Generally speaking, the spread is optimal when the RMSE for the testing samples is the least. The spreads between 0.02 and 0.03 with an interval of 0.0005 were used to find the minimum RMSE. By trial and error, we found that when the spread was 0.0265, the RMSE for the testing samples is the least (Fig 3). Therefore, we constructed the GRNN with the spread 0.0265. The forecasted incidence values of ARIMA model from January 2012 to December 2012 were used as the input of the constructed ARIMA-GRNN hybrid model. The prediction incidence values were forecasted with the constructed hybrid model.

### ARIMA-NARNN Hybrid Model

**Fig 4:**
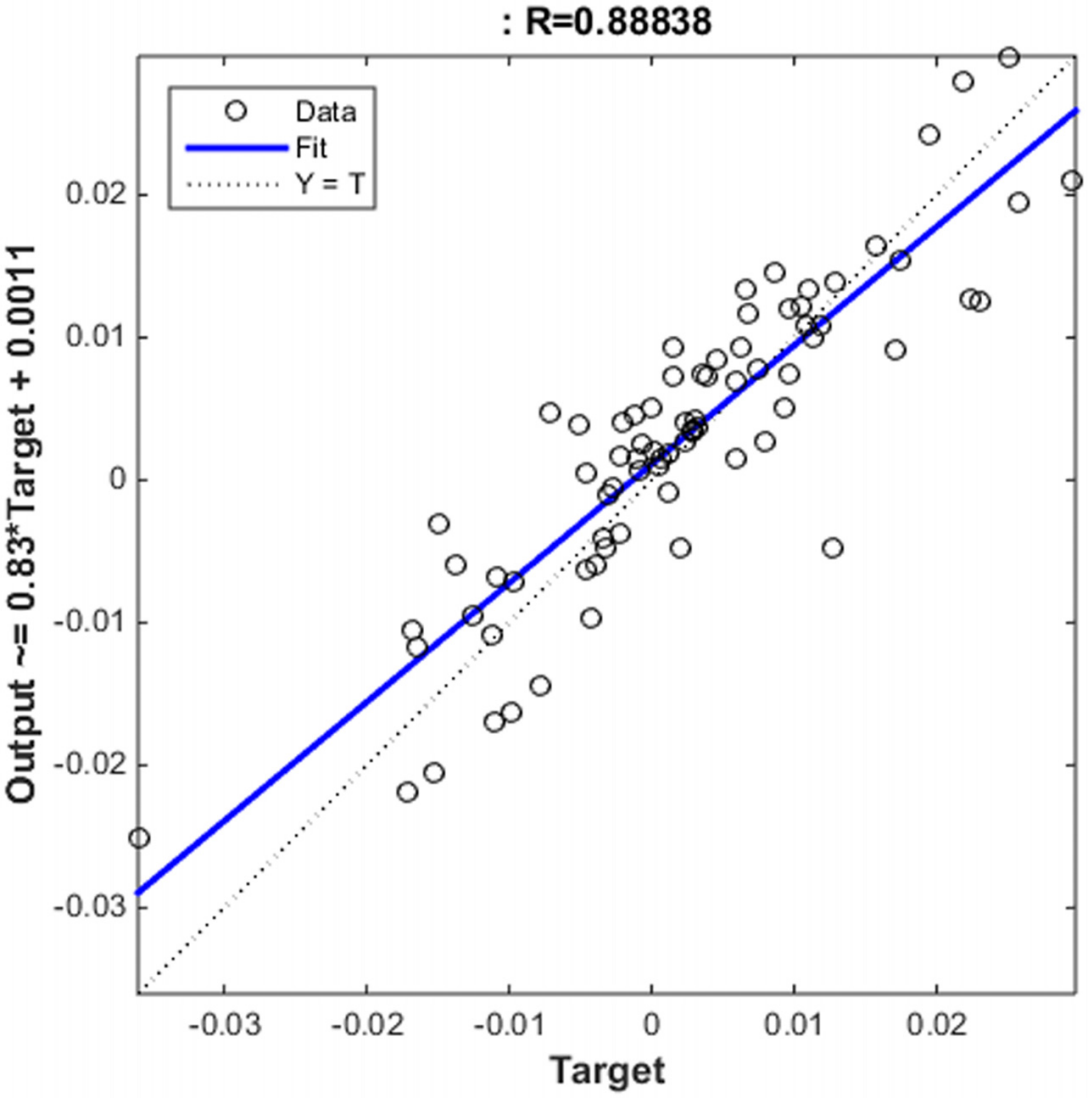
The plot of actual residual values and NARNN model fitted values.

**Fig 5:**
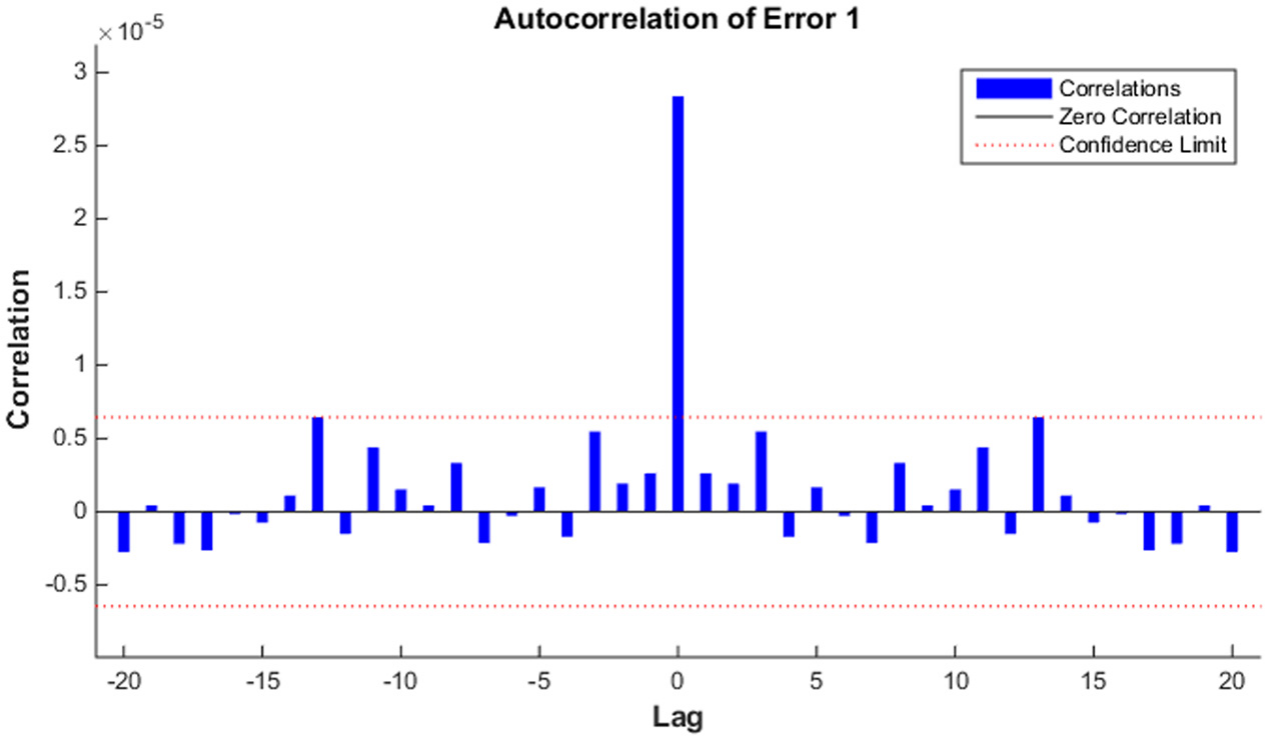
The error autocorrelation plot of target series. The correlations, except for the one at zero lag, fall approximately within the 95% confidence limits around zero, so the model seems to be adequate.

**Fig 6:**
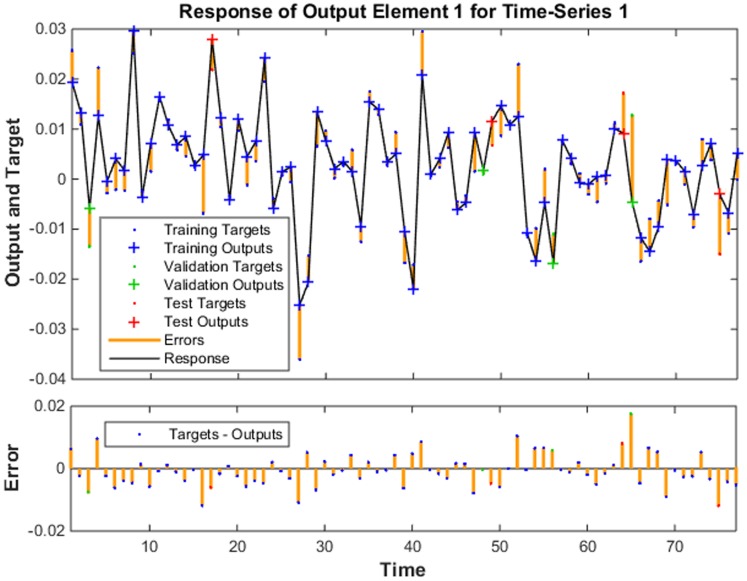
The time series response plot of target series. This plot displays the inputs, targets and errors versus time. In the training, validation and testing samples the errors were small. We assured that the model was suitable.

Residual values between February 2005 and December 2011 were used to construct the NARNN model. By trial and error, when the number of hidden units and delays were 35 and 6, respectively, the NARNN model was optimal. The plot of actual residual values and NARNN model fitted values is shown in Fig 4. The R value was 0.8884 greater than 0.8. The error autocorrelation function plot is shown in Fig 5. The correlations, except for the one at zero lag, fall approximately within the 95% confidence limits around zero, so the model seems to be adequate. Time series response plot is displayed in Fig 6. This plot displays the inputs, targets and errors versus time. In the training, validation and testing samples the errors were acceptable. The adjusted residuals from January 2012 to December 2012 were forecasted with the constructed ARIMA- NARNN hybrid model.

### Comparison of Modeling and Forecasting Performance

**Fig 7:**
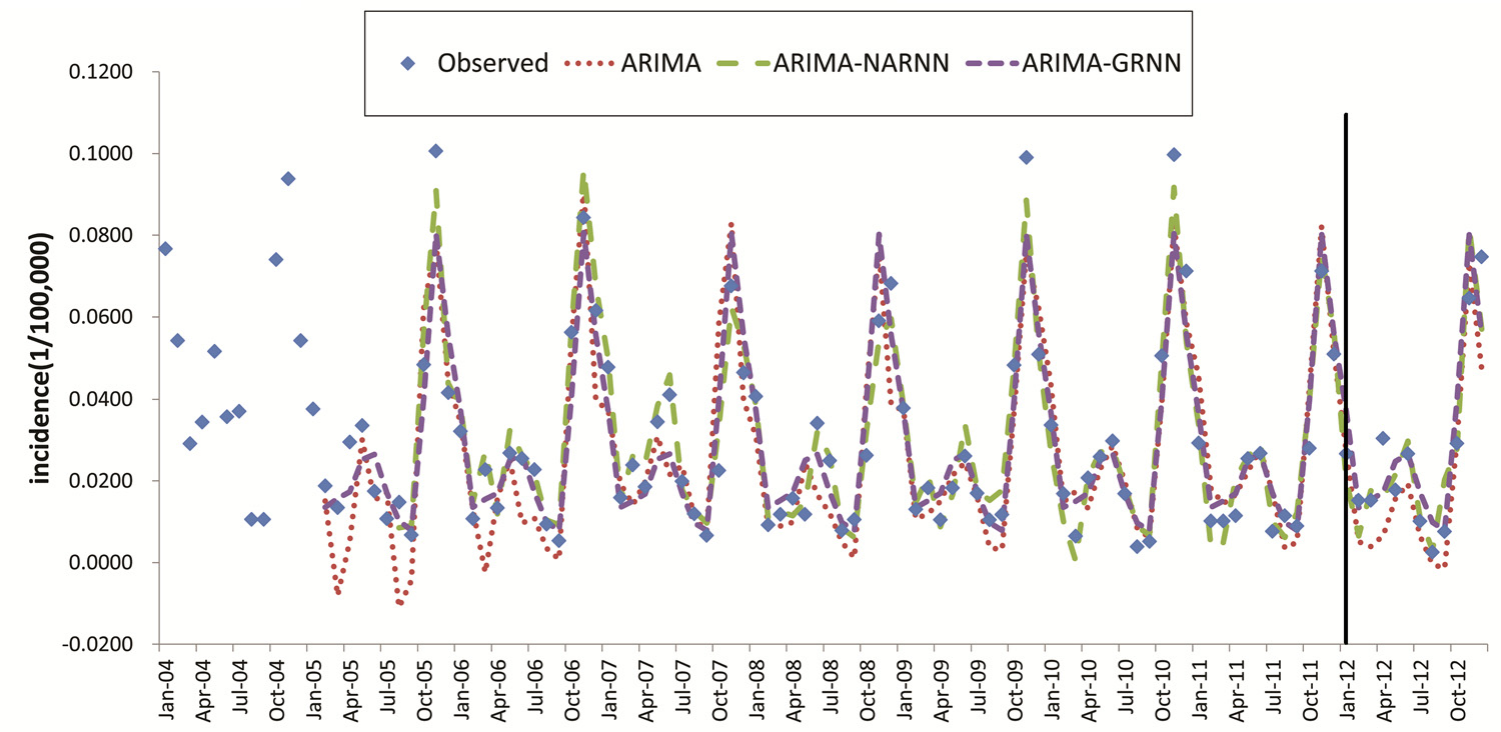
The observed HFRS incidence and modeling and forecasting values simulated by ARIMA, ARIMA-NARNN and ARIMA-GRNN model. The data set was divided into modeling and forecasting data set with a vertical line; the left is the modeling stage, and the right is the forecasting stage.

The fitting and forecasting incidence values of different methods are displayed in Fig 7. All of the three models fitted and predicted the seasonal fluctuation well. Incidence values for 2012 forecasted by three models are shown in Table 3. The performances of three models were compared in Table 4. Among the three models, the MSE, MAE and MAPE of ARIMA-NARNN hybrid model was the lowest both in modeling stage and forecasting stage. As for the ARIMA-GRNN hybrid model, although the MSE, MAE and MAPE of modeling performance and the MSE and MAE of forecasting performance were less than the ARIMA model, but the MAPE of forecasting performance was bigger than the ARIMA model.

**Table 3:** Incidence values for 2012 forecasted by ARIMA, ARIMA-GRNN and ARIMA-NARNN model.

Time	Observed	ARIMA	ARIMA-GRNN	ARIMA-NARNN
January	0.0266	0.0272	0.0372	0.0202
February	0.0152	0.0051	0.0134	0.0066
March	0.0152	0.0040	0.0154	0.0176
April	0.0304	0.0066	0.0172	0.0159
May	0.0177	0.0156	0.0248	0.0215
June	0.0266	0.0193	0.0266	0.0297
July	0.0101	0.0065	0.0172	0.0111
August	0.0025	-0.0004	0.0099	0.0030
September	0.0076	-0.0014	0.0079	0.0203
October	0.0291	0.0302	0.0400	0.0279
November	0.0646	0.0733	0.0802	0.0818
December	0.0747	0.0472	0.0556	0.0571

**Table 4:** Comparison of the modeling and forecasting performance of ARIMA, ARIMA-GRNN and ARIMA-NARNN model.

Model	Modeling performance			Forecasting performance		
	MSE	MAE	MAPE	MSE	MAE	MAPE
ARIMA	1.3857	0.0089	0.4219	1.4866	0.0090	0.4859
ARIMA-GRNN	0.6785	0.0063	0.2815	0.9746	0.0078	0.4899
ARIMA-NARNN	0.2836	0.0042	0.2199	0.9401	0.0074	0.3566

Note: ALL MSE values should be multiplied by 10^−4^.

## Discussion

In this study, we constructed two hybrid models based on traditional ARIMA model to fit and forecast the HFRS incidence, one combined with GRNN and the other combined with NARNN. As far as we know, this is the first study to use the two hybrid models to fit and predict the HFRS incidence in China.

Time series analysis is used to deal with the probabilistic and structural inference about a sequence of data evolving through time. ARIMA model is one of the most commonly used methods in time series analysis because the Box-Jenkins methodology is a well-developed method in the modeling process and we can obtain the exact model parameters. ARIMA model is one of the most effective linear models for seasonal time series forecasting [10]. In this study, the ARIMA model well captured the seasonal fluctuation of HFRS, but the performances of modeling and forecasting were not satisfactory. As is known to all, the occurrence of HFRS is affected by a lot of factors such as animal reservoir, climatic and socioeconomic variations [22, 23]. We supposed that nonlinear relations may exist among the monthly HFRS incidences and the ARIMA model cannot extract the full relationship efficiently. In order to improve the model, we decided to construct hybrid models combining the ARIMA model and ANNs. ANNs are based on present understanding of the biological nervous system the ability to learn through example [24] and have been widely used as efficient methods in modeling nonlinear and dynamic systems [25]. GRNN is one of the widely used static neural networks. Compared with the back propagation neural network GRNN has advantages of fast training time, easy network parameter and great stability in the training stage [14]. Most of the published studies focusing on ARIMA-GRNN hybrid model got the same result that the MSE, MAE and MAPE of ARIMA-GRNN model were less than single ARIMA model both in modeling and forecasting stage [14–16]. In this study, the MSE, MAE and MAPE of ARIMA-GRNN model were less than single ARIMA model in modeling stage, and the MSE and MAE of ARIMA-GRNN model were also less than single ARIMA model in forecasting stage, but the MAPE of ARIMA-GRNN model did not improve. The MAPE of ARIMA-GRNN and ARIMA model was 0.4899 and 0.4859, respectively. The GRNN is based on nonparametric kernel regression. The appropriate underlying regression surface is automatically extracted from the data. The spread factor plays a main role in function approximation. A smaller spread factor leads to a steeper radial basis function, then the fitting values will more close to the real values, but the generalization ability will be weak. On the other hand, a bigger spread factor will make the fitting curve smoother, but the performance of fitting will be poor. In this study, we just need to select the appropriate spread with the method proposed by Specht [20] in modeling training. Two samples were selected randomly in the modeling data set as testing samples to select the optimal spread factor. The series of HFRS incidence values has a strong seasonality trend and it is necessary to use the time values as one input of GRNN. In the study, the estimated monthly incidence values of ARIMA and corresponding time values were used as two inputs of GRNN model. The performance of ARIMA-GRNN improved greatly than only used the estimated monthly incidence values of ARIMA as input of GRNN.

Dynamic neural networks are good at time series prediction. NARNN is a dynamic neural network, and is always more powerful than static neural networks because it has memory and can be trained to learn time-varying patterns [12]. In the study, the performance of dynamic neural network NARNN was better than static neural network GRNN. During the training of the network we could obtain the true output, in order to obtain a more accurate network, we used the open loop architecture, in which the true output was used instead of feeding back the estimated output. With the ARIMA-NARNN hybrid model, the linear and nonlinear components of the HFRS incidence in Jiangsu province China were extracted efficiently and the modeling and forecasting performance increased sharply.

Some limitations in this study should be taken into account. Firstly, we just conducted time series analysis without considering the factors affected the occurrence of HFRS such as animal reservoir, climatic and socioeconomic variations. Secondly, since the CNSS was established in 2004, we could only obtain the data from 2004 to 2012. More data can improve the efficacy of these models. Thirdly, this study only focused on Jiangsu province. Whether these models are suitable for other epidemic places or other infectious diseases needs further study.

## Conclusions

On the whole, the ARIMA-NARNN hybrid model is superior to the ARIMA model and ARIMA-GRNN hybrid model in fitting and forecasting the incidence of HFRS in Jiangsu Province, China. Developing and applying the ARIMA-NARNN hybrid model is an effective method to make us better understand the epidemic characteristics of HFRS and could be helpful to the prevention and control of HFRS. It is necessary to apply the hybrid model in other epidemic places or other infectious diseases to detect the degree of generalization of the findings obtained in this study.

## Supporting Information

S1 File Monthly HFRS incidence series of Jiangsu province in China from January 2004 to December 2012. (XLSX)
